# Effect of obesity reduction on preservation of heart function and attenuation of left ventricular remodeling, oxidative stress and inflammation in obese mice

**DOI:** 10.1186/1479-5876-10-145

**Published:** 2012-07-11

**Authors:** Hui-Ting Wang, Chu-Feng Liu, Tzu-Hsien Tsai, Yung-Lung Chen, Hsueh-Wen Chang, Ching-Yen Tsai, Steve Leu, Yen-Yi Zhen, Han-Tan Chai, Sheng-Ying Chung, Sarah Chua, Chia-Hung Yen, Hon-Kan Yip

**Affiliations:** 1 Department of Emergency Medicine, Kaohsiung Chang Gung Memorial Hospital and Chang Gung University College of Medicine, Kaohsiung, Taiwan; 2 Division of cardiology, Department of Internal Medicine, Kaohsiung Chang Gung Memorial Hospital and Chang Gung University College of Medicine, Kaohsiung, Taiwan; 3 Department of Biological Sciences, National Sun Yat-Sen University, Kaohsiung, Taiwan; 4 Institute of Molecular Biology, Academia Sinica, Taipei, Taiwan; 5 Center for Translational Research in Biomedical Sciences, Kaohsiung Chang Gung Memorial Hospital and Chang Gung University College of Medicine, Kaohsiung, Taiwan; 6 Department of Biological Science and Technology, National Pingtung University of Science and Technology, Pingtung, Taiwan

**Keywords:** Obesity, Inflammation, Oxidative stress, Apoptosis, Fibrosis

## Abstract

**Background:**

Obesity is an important cardiovascular risk factor. This study tested the effect of obesity reduction on preserving left ventricular ejection fraction (LVEF) and attenuating inflammation, oxidative stress and LV remodeling in obese mice.

**Methods and results:**

Eight-week-old C57BL/6 J mice (n=24) were equally divided into control (fed a control diet for 22 weeks), obesity (high-fat diet, 22 weeks), and obese reduction (OR) (high-fat diet, 14 weeks; then control diet, 8 weeks). Animals were sacrificed at post 22-week high-fat diet and the LV myocardium collected. Heart weight, body weight, abdominal-fat weight, total cholesterol level and fasting blood glucose were higher in obesity than in control and OR (all p<0.001). Inflammation measured by mRNA expressions of IL-6, MMP-9, PAI-1 and leptin and protein expression of NF-κB was higher, whereas anti-inflammation measured by mRNA expressions of adiponectin and INF-γ was lower in obesity than in control and OR (all p<0.003). Oxidative protein expressions of NOX-1, NOX-2 and oxidized protein were higher, whereas expression of anti-oxidant markers HO-1 and NQO-1 were lower (all p<0.01); and apoptosis measured by Bax and caspase 3 was higher, whereas anti-apoptotic Bcl-2 was lower in obesity as compared with control and OR (all p<0.001). The expressions of fibrotic markers phosphorylated Smad3 and TGF-β were higher, whereas expression of anti-fibrotic phosphorylated Smad1/5 and BMP-2 were lower (all p<0.02); and LVEF was lower, whereas the LV remodeling was higher in obesity than in control and OR (all p<0.001).

**Conclusion:**

Impaired LVEF, enhanced LV remodeling, inflammation, fibrosis, oxidative stress and apoptosis were reversed by reduction in mouse obesity.

## Introduction

Obesity is a major global problem that affects health and quality of life [1-3]. Age-related increases in obesity are associated with a notably higher prevalence of metabolic disease, several common cancers, and numerous other cardiovascular diseases [4-6]. Obesity has been also shown to greatly increase functional limitations and disability [4-8]. Additionally, obesity adversely affects the circulatory system resulting in endothelial dysfunction, which promotes systemic hypertension, coronary artery disease, and vascular calcification [9-12]. There is thus an urgent need to develop comprehensive interventions to mitigate obesity, especially in older adults.

Obesity is often characterized by increased local and systemic oxidative stress and exacerbated inflammatory reactions accompanied by infiltration of immune cells into adipocytes[13-15]. Moreover, abundant data suggest that oxidative stress and inflammatory signaling are not only interrelated, but that their upregulation can lead to inhibition in insulin response to glucose and also contribute to atherosclerosis, cardiovascular diseases and their associated features [5,13-19]. Previous study [20] has revealed that oxidative stress and inflammation contributed to the generation of cellular apoptosis and fibrosis in setting of dilated cardiomyopathy. The study [20] has further identified that enhancement of these biomarkers played an essential role on deteriorating the heart function. However, whether these biomarkers are also up-regulated in setting of obesity remains uncertain.

Although current scientific data emphasizes that obesity is a risk factor for cardiovascular disease and highlights the interplay between oxidative stress and inflammation and obesity/diabetes onset [4-19], the relationship between obesity and heart function is poorly understood. Some clinical observation studies have demonstrated that LV systolic and diastolic functions are impaired in patients with metabolic syndrome even if they have normal LVEF [21]. However, other studies have revealed that obesity is only associated with concentric LV remodeling without change in ejection [22]. Pharmacologic and non-pharmacologic interventions that target weight-loss benefits are infrequently reported [23-25], especially with respect to investigation of the benefit of obesity reduction on LV function. Using a high fat diet-induced mouse model of obesity, the aim of this study was to test the hypotheses that, in obese mice: 1) inflammation, oxidative stress, fibrosis and apoptosis were significantly enhanced in the LV myocardium; and 2) LV function was significantly impaired, whereas LV remodeling was remarkably increased in obese mice; and 3) molecular-cellular perturbations, LV dysfunction and LV remodeling were significantly reversed after reduction in obesity.

## Materials and methods

### Ethics

All animal experimental procedures were approved by the Institute of Animal Care and Use Committee at Kaohsiung Chang Gang Memorial Hospital and performed in accordance with the Guide for the Care and Use of Laboratory Animals (NIH publication No. 85–23, National Academy Press, Washington, DC, USA, 1996).

### Animal model of obesity

**Figure 1:**
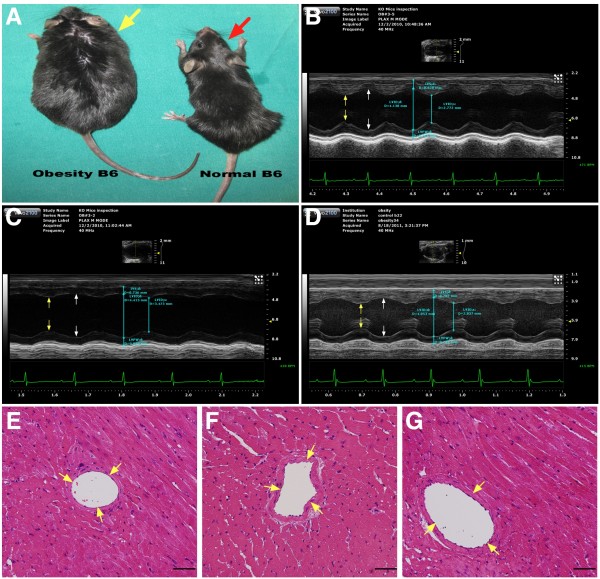
**General appearance, illustration of M-mode echocardiography and pathological findings in among three groups of animals (n = 8 for each group). A) **Comparison of general appearance of obesity mouse (yellow arrow) and normal B6 mouse (red arrow) at the study period. **B) to D)** Showing both systolic (yellow arrows) and diastolic (white arrows) dimensions were notably dilated in obesity mouse (**C**) than in normal control (**B**) that were revised by obesity reduction (**D**). **E) to G)** The results of H & E stain showed no obstructive coronary artery disease was noted among normal (**E**), obesity (**F**) and obesity reduction (**G**) animals.

Eight-week-old male C57BL/6 J mice (n = 24), weighing 22–24 g, (Charles River Technology, BioLASCO, Taiwan), were fed with a high-fat diet (45 kcal% fat; Research Diets) to create a diet-induced obesity model. According to the literature [26] and the instructions from the diet manufacturer, successful obesity induction is defined as an increase in mouse body weight of more than 35 % after feeding with the diet for 13 weeks. After feeding with the high fat diet for 12 weeks, 75 % mice in our study fit the criteria of obesity (Figure 1-A).

Sixteen of the obese mice were then equally divided into two groups: obesity that were continuously fed with a high fat diet for further 10 weeks, and obese reduction (i.e., body-weight reduction) that were continuously fed with a high fat diet for further 2 weeks, followed by standard mouse chow (i.e., the control diet) for a subsequent 8 weeks. Another group (normal control) of age-matched C57BL/6 J mice (n = 8) were fed with for the same duration (22-weeks) with a control diet that was also purchased from the same company (Research Diets). This group served as untreated controls.

### Functional assessment by echocardiography

All animals underwent transthoracic echocardiography under anesthesia in a supine position at the beginning and end of the study. The procedure was performed by an animal cardiologist blind to the experimental design using a VisualSonics ultrasound machine (Vevo, 2100). M-mode standard two-dimensional (2DE) left parasternal-long axis echocardiographic examination was conducted. Left ventricular internal dimensions [end-systolic diameter (ESD) and end-diastolic diameter (EDD)] were measured according to the American Society of Echocardiography leading-edge method using at least three consecutive cardiac cycles (Figure 1-B, 1-C, 1-D). LVEF was calculated as follows: LVEF (%)=[(LVEDD^3^-LVEDS^3^)/LVEDD^3^] × 100

### Real-time quantitative PCR analysis

Real-time reverse transcription polymerase chain reaction (RT-qPCR) was conducted using LighCycler TaqMan Master (Roche, Germany) in a single capillary tube according to the manufacturer’s guidelines for individual component concentrations. Forward and reverse primers (Table 1) were each designed in a different exon of the target gene sequence, eliminating the possibility of amplifying genomic DNA. The negative control (single primer test and templateless) was also performed for each assay.

**Table 1:** Primer Used for Real-Time PCR Amplification

**Gene**	**GenBank accession number**	**Forward Primer (5 ´-3 ´)**	**PCR product size (bp)**
		**Reverse Primer (5 ´-3 ´)**	
IL-6	NM_012589.1	CCCTTCAGGAACAGCTATGAA	74
		ACAACATCAGTCCCAAGAAGG	
MMP-9	NM_031055.1	CCTCTGCATGAAGACGACATAA	64
		GGTCAGGTTTAGAGCCACGA	
Leptin	NM_008493.3	CAGGATCAATGACATTTCACACA	145
		GCTGGTGAGGACCTGTTGAT	
IL-1β	NM_214055.1	CCTGGACCTTGGTTCTCTGA	61
		CCAGAGCTGGTGAGAGATTTG	
Adiponectin	NM_009605.4	GGAGAGAAAGGAGATGCAGGT	109
		CTTTCCTGCCAGGGGTTC	
PAI-1	NM_213910.1	GCCCTGGTGACTCATTTCA	65
		CTCTCACGTGTCCACTGCTC	
IFN-γ	NM_008337.3	ATCTGGAGGAACTGGCAAAA	89
		TTCAAGACTTCAAAGAGTCTGAGGTA	
ET-1	M64711.1	TGTCTACTTCTGCCACCTGGA	69
		CCTAGTCCATACGGGACGAC	
AT-1R	NM_030985.4	CACCCGATCACCGATCAC	110
		CAGCCATTTTATACCAATCTCTCA	
Bax	NM_017059.1	GTGAGCGGCTGCTTGTCT	107
		GACTCCAGCCACAAAGATGG	
Caspase 3	NM_009810.2	TGAAGACATTTTGGAATTAATGGA	90
		TCACCATGGCTTAGAATCACA	
Bcl-2	NM_016993.1	GGGATGCCTTTGTGGAACT	82
		CTGAGCAGCGTCTTCAGAGA	
HO-1	NM_001004027	CATTCAGCTGTTTGAGGAGGT	64
		CGAGGGTCTCTGGTCCTTAGT	
NQO-1	NM_001159613	CCGAGAAGACGTCCTTCAAT	96
		TGGCATACAGGTCCGACAC	
α-actin	DQ452569	CTGGACTTCGAGCAGGAGAT	69
		GGCAGCTCGTAGCTCTTCAT	

IL = interleukin; MMP = matrix metalloproteinase; PAI = lasminogen activator inhibitor; IFN = interferon; ET = endothelin; AT-1R = angiotensin II type 1 receptor; HO = heme oxygenase; NQO = NAD(P)H quinone oxidoreductase.

Total RNA was extracted using a spin column-based RNA extraction kit (RNeasy Fibrous Tissue Mini Kit, Qiagen) according to protocols provided by manufacturer. Reverse transcriptions were performed with the Transcriptor First Strand cDNA Synthesis Kit (Roche). Briefly, 1 μg of total RNA was mixed with 50 pmole oligo dT and then incubated at 65 °C for 10 minutes. After incubation on ice for 5 minutes, 4 μL of 5× reverse transcriptase reaction buffer, 0.5 μL of RNase inhibitor (40 U/μl), 2 μL of dNTP (10 mM for each), and 0.5 μL of reverse transcriptase (20 U/μL) were added into tubes containing hybridized RNA-oligo dT mixtures. RT reactions were carried out at 55 °C for 30 minutes. PCRs were performed on a Light Cycler (Roche Molecular Biochemicals). Each reaction was carried out with 1 μL of cDNA, 5 μL of 2× reaction mixtures (Maxima Probe qPCR Master Mix), 0.15 μL or each primer (20 μM), 0.2 μL of probe, and 3.5 μL of sterile distilled water. Reactions were performed by incubating at 95 °C for 10 minutes, following with 45 cycles of 95 °C for 10 sec, 60 °C for 30 sec, and 72 °C for 1 sec. Analysis of melting curves and determination of threshold cycle (Ct) were performed by the Light Cycler instrument software provided by Roche.

### Isolation of mitochondria from LV myocardium

The procedure and protocol of mitochondrial isolation were described in details in our previous report [20]. Briefly, the LV myocardium was excised and washed with buffer A (100 mM Tris–HCl, 70 mM sucrose, 10 mM EDTA, and 210 mM mannitol, pH 7.4). Samples were minced finely in cold buffer A and incubated for 10 minutes at 4 °C. All samples were homogenized in an additional 3 mL of buffer A using a motor-driven grinder. The homogenate was centrifuged twice at 700 *g* for 10 minutes at 4 °C. The supernatant was centrifuged again at 8,500 *g* for 15 minutes, and the pellets were washed with buffer B (10 mM Tris–HCl, 70 mM sucrose, 1 mM EDTA, and 230 mM mannitol, pH 7.4). The mitochondria-rich pellets were collected and stored at −70 °C.

### Western blot analysis

The procedure and protocol of protein extracts from LV myocardium were described in details in our previous report [20]. Briefly, equal amounts (10–30 μg) of protein extracts from LV myocardium of the animals were loaded and separated by SDS-PAGE using 12 % acrylamide gradients. The membranes were incubated with monoclonal antibodies against phospho-Smad 3 (1:1000, Cell Signaling ), transforming growth factor (TGF)-β (1:500, Abcam ), phospho-Smad1/5 (1:1000, Cell Signaling ), bone morphogenic protein (BMP)-2 (1:500, Abcam), NADPH oxidase (NOX)-1 (1: 1000, Abcam), NOX-2 (1: 1000, Abcam), NAD(P)H Quinone Oxidoreductase (NQO 1) (1: 1000, Abcam), Heme Oxygenase (HO-1) (1: 1000, Abcam), nuclear factor (NF)-κB (1: 250, Abcam), Bax (1: 1000, Abcam), caspase 3 (1:1000, Cell Signaling) and Bcl-2 (1: 200, Abcam). Signals were detected with HRP-conjugated goat anti- mouse or goat anti-rabbit IgG.

Oxyblot protein analysis was carried out using Oxyblot Oxidized Protein Detection Kit from Chemicon (S7150). Proteins were transferred to nitrocellulose membranes which were then incubated in the primary antibody solution (anti-DNP 1: 150) for two hours, followed by incubation with second antibody solution (1:300) for one hour at room temperature. The washing procedure was repeated eight times within 40 minutes. Immunoreactive bands were visualized by enhanced chemiluminescence (ECL; Amersham Biosciences) which was then exposed to Biomax L film (Kodak). For quantification, ECL signals were digitized using Labwork software (UVP). For oxyblot protein analysis, a standard control was loaded on each gel.

### Statistical analysis

Quantitative data are expressed as means ± SD. Statistical analysis was adequately performed by ANOVA followed by Bonferroni’s multiple-comparisons post hoc test. Statistical analysis was performed using SAS statistical software for Windows version 8.2 (SAS institute, Cary, NC). A P value of less than 0.05 was considered statistically significant.

## Results

### Baseline characteristics, laboratory and echocardiography data, and histology of left ventricle (n=8 for each group)

Data were summarized in Table 2. Initial body weight and fasting blood sugar did not differ among the normal controls (control), the obese mice (obesity) and the obesity induction followed by subsequent body weight reduction group (obese reduction). However, the final body weight was substantially higher in the obesity than in control and obese reduction (p<0.0001). There was no difference in body weight between control and obese reduction. Final fasting blood sugar, abdominal fat weight and serum cholesterol were considerably higher in obesity than in control and obese reduction, and significantly higher in obese reduction than in control (all p <0.001). Total heart weight and ratio of heart weight to tibial bone length were considerably higher in obesity than in control and obese reduction (p<0.001), but there was no significant difference between the later two groups.

**Table 2:** The Baseline Characteristics, Laboratory Findings and Echocardiography Results

**Variables**	**Control**	**Obesity**	**Obese reduction**	P value*
	**(n=8)**	**(n=8)**	**(n =8)**	
Initial body weight (g)	26.0 ± 0.72	25.7 ± 0.66	25.7 ± 0.87	0.675
Final body weight (g)	34.4 ± 1.48^a^	43.9 ± 1.55^b^	35.4 ± 2.21^a^	<0.0001
Heart weight (g)	0.14 ± 0.01^a^	0.19 ± 0.02^b^	0.15 ± 0.02^a^	<0.0001
Ratio of heart weight to tibial bone length	0.070 ± 0.001^a^	0.095 ± 0.002^b^	0.075 ± 0.017^a^	<0.001
Abdominal fat weight (g)	1.66 ± 0.41^a^	4.09 ± 0.65^b^	2.35 ± 0.08^c^	<0.0001
Total cholesterol (mg/dl)†	136.3 ± 11.8^a^	232.8 ± 7.53^b^	188.8 ± 9.45^c^	<0.001
Initial fasting blood glucose level (mg/dl)†	98.3 ± 9.0	103 ± 8.6	101.3 ± 9.4	0.688
Final fasting blood glucose level (mg/dl)†	142.5 ± 18.3^a^	307.5 ± 74.7^b^	181.2 ± 19.7^c^	<0.0001
Echocardiograph (baseline)				
Thickness of inter-ventricular septum (mm)	0.83 ± 0.09	0.82 ± 0.08	0.818 ± 0.098	0.616
Thickness of posterior wall (mm)	0.76 ± 0.09	0.77 ± 0.11	0.776 ± 0.089	0.720
Left ventricular end-diastolic dimension (mm)	3.93 ± 0.26	4.10 ± 0.10	4.108 ± 0.083	0.117
Left ventricular end-systolic dimension (mm)	2.63 ± 0.23	2.71 ± 0.21	2.69 ± 0.170	0.809
Fractional shortening (%)	34.4 ± 2.01	32.7 ± 1.98	32.98 ± 1.64	0.240
Left ventricular ejection fraction (%)	64.0 ± 2.49	62.5 ± 2.43	62.27 ± 2.04	0.455
Echocardiograph (at end of study period)				
Thickness of inter-ventricular septum (mm)	0.86 ± 0.08	0.87 ± 0.06	0.859 ± 0.07	0.789
Thickness of posterior wall (mm)	0.77 ± 0.03	0.79 ± 0.08	0.791 ± 0.09	0.916
Left ventricular end-diastolic dimension (mm)	4.18 ± 0.16^a^	4.456 ± 0.17^b^	4.22 ± 0.19^a^	0.016
Left ventricular end-systolic dimension (mm)	2.78 ± 0.13^a^	3.462 ± 0.49^b^	3.09 ± 0.33^c^	<0.001
Fractional shortening (%)	33.44 ± 0.85^a^	22.82 ± 1.15^b^	27.86 ± 1.61^c^	<0.001
Left ventricular ejection fraction (%)	62.66 ± 1.43^a^	46.04 ± 1.95^b^	54.05 ± 5.41^c^	<0.001

* performed by ANOVA; means with different letters (a, b, c) indicate significant difference (at level 0.05) by Bonferroni’s multiple-comparisons post hoc test.

† value was observed after 12 fasting in the mice.

The transthoracic echocardiographic findings showed that the thickness of interventricular septum and posterior wall prior to and at the end of the study period were similar among all the groups of animals. Additionally, before induction of obesity, the LVEDD, LVESD, LVEF and the LV fractional shortening (%) showed no difference among the three groups. However, by the end of study period, the LVEDD and LVESD were significantly higher in obesity than in control and obese reduction, and the LVESD was notably higher in obese reduction than in control (all p<0.02), whereas the LVEDD did not differ between these two groups. Conversely, LVEF and the LV fractional shortening were significantly lower in obesity than the control and obese reduction, and significantly lower in obese reduction than in the control (all p<0.001).

The histological findings (i.e., H&E staining) of LV myocardium revealed no atherosclerotic obstructive coronary artery disease among three groups of the animals.

### Protein and mRNA expressions of inflammatory and anti-inflammatory biomarkers in the LV myocardium at the End of the study period (n=8 for each group)

**Figure 2:**
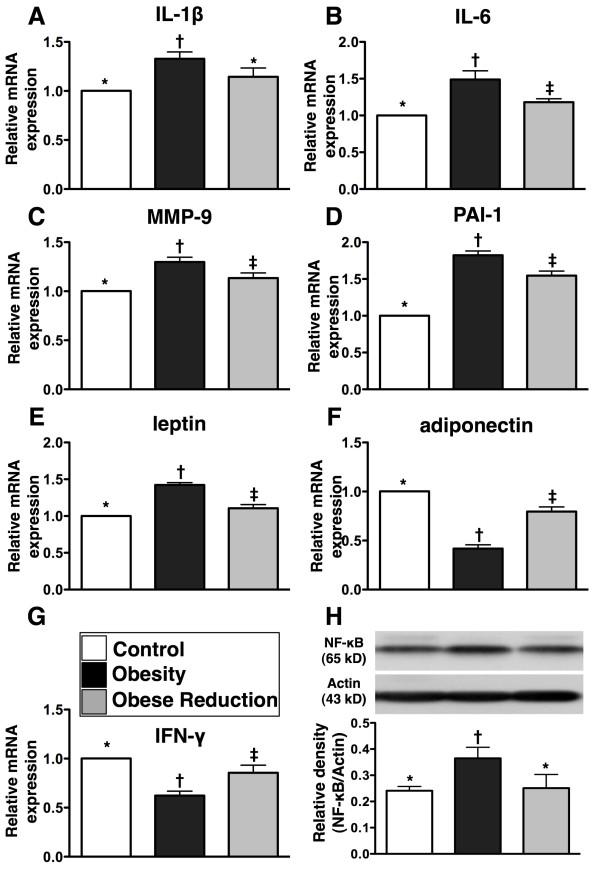
**Inflammatory and anti-inflammatory biomarkers in left ventricular myocardium at the end of study period (n = 8 for each group). A) to E) **The mRNA expressions of interleukin (IL)-1β, IL-6, matrix metalloproteinase (MMP)-9, plasminogen activator inhibitor (PAI)-1 and leptin, indices of inflammation, were significantly higher in obesity group than in control and obese-reduction groups, and except for IL-1β, other were significantly higher in obese-reduction group than in control group. * vs. † vs. ‡, or * vs. †, p<0.001. **F) and G)** The mRNA expressions of adiponectin and interferon (INF)-γ, two indicators of anti-inflammatory and immunomodulatory biomarkers, were significantly lower in obesity group than in control and obese-reduction groups, and significantly lower in obese-reduction group than in control group. * vs. † vs. ‡, p<0.001. **H)** The nuclear factor (NF)-κB protein expression was significantly higher in obesity group than in control and obese-reduction groups, but it showed no difference in the later two groups. * vs. †, p<0.01. All statistical analyses using one-way ANOVA, followed by Bonferroni multiple comparison post hoc test. Symbols (*, †, ‡) indicate significance (at 0.05 level) among **A)** to **H).**

To determine the effect of obesity and obesity reduction on inflammatory reaction in the LV Myocardium, RT-PCR and western blot were performed (Figure 2). The results showed that mRNA expressions of interleukin (IL)-1β, IL-6, matrix metalloproteinase (MMP)-9, plasminogen activator inhibitor (PAI-1) and leptin, five indices of inflammation, were significantly higher in the obesity than in control and obese reduction, and all of these parameters except for IL-1β were significantly higher in obese reduction than in the control (all p <0.001). Additionally, the protein expression of NF-κB, also an index of inflammation was significantly higher in obesity than in control and obese reduction (p<0.01), but it showed no difference in obese reduction as compared with the control. On the other hand, the mRNA expressions of adiponectin, an anti-inflammatory biomarker, and interferon (INF)-γ, an immunomodulatory biomarker, were remarkably lower in obesity than in control and obese reduction, and significantly lower in obese reduction than in control (all p<0.001).

### Protein and mRNA expressions of markers of vasoconstriction and oxidative stress in LV myocardium at the End of study period (n=8 for each group)

**Figure 3:**
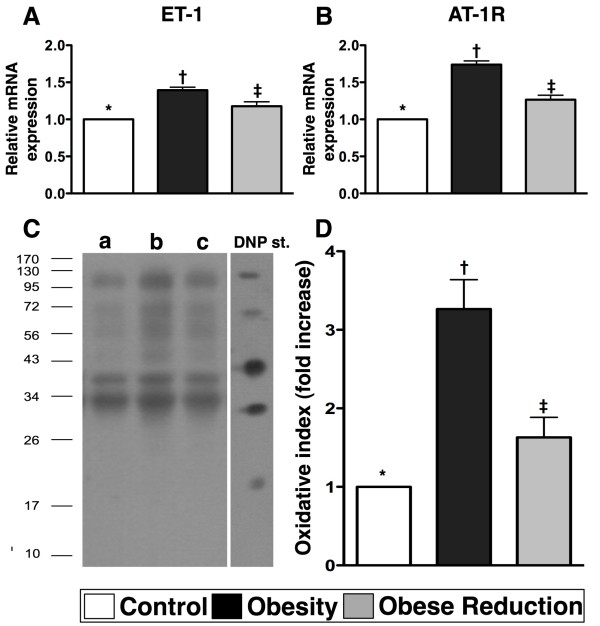
**The mRNA and protein expressions of vasoconstriction and oxidative-stress markers (n = 8 for each group) .A) and B) **The mRNA expressions of endothelin (ET)-1 and angiotensin II type I receptor (AT-1R), two indicators of vasoconstrictions/impairment of microcirculation, were remarkably increased in obesity group than in control and obese-reduction groups, and notably increased in obese-reduction group than in control group. * vs. † vs. ‡, p<0.001. **C)** Showing the Western blot of oxidized protein (Note: Right lane and left lane shown on left lower panel represent control oxidized molecular protein standard and protein molecular weight marker, respectively). DNP = 1–3 dinitrophenylhydrazone. a = normal, b = obesity, c = obese reduction. **D)** Showing significantly higher oxidative index, protein carbonyls, in obesity group than in normal and obese-reduction groups, and significantly higher in obese-reduction group than in control group. * vs. † vs. ‡, p<0.001. All statistical analyses using one-way ANOVA, followed by Bonferroni multiple comparison post hoc test. Symbols (*, †, ‡) indicate significance (at 0.05 level) among **A), B),** and **D).**

**Figure 4:**
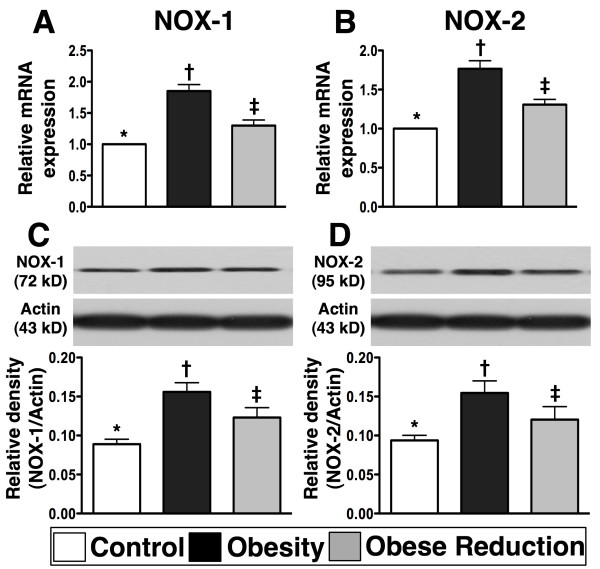
**The mRNA and protein expressions of reactive oxygen species (ROS) (n = 8 for each group). A & B) and C & D) **showing the gene (**A, B**) and protein (**C, D**) expressions of NADPH oxidase (NOX)-1 and NOX-2, two indexes of ROS generation, were remarkably higher in obesity group than in normal and obese-reduction groups, and notably higher in obese-reduction group than in normal group. For A & B) * vs. † vs. ‡, p<0.001; For **C & D)**. * vs. † vs. ‡, p<0.01. All statistical analyses using one-way ANOVA, followed by Bonferroni multiple comparison post hoc test. Symbols (*, †, ‡) indicate significance (at 0.05 level) among **A) to D).**

To elucidate the effects of obesity and reduction in obesity on vasoconstriction and oxidative stress, RT-PCR and western blot were performed (Figures 3 and 4). The mRNA expression of endothelin (ET)-1, an indicator of endothelial dysfunction, was markedly increased in the obesity in comparison with control and obese reduction, and was also significantly increased in obese reduction in comparison with control (Figure 3) (p<0.001). The mRNA expression of angiotensin II type I receptor (AT-1R) (Figure 3), an indicator of vasoconstriction and reactive oxygen species (ROS), showed the same pattern as ET-1 among the three groups (Figure 3) (p<0.001). Furthermore, the protein expressions of oxyblot (i.e., protein carbonyls) (Figure 3), an index of oxidation, and mRNA and protein expressions of NOX-1 and NOX-2 (Figure 4), two indices of ROS, were significantly increased in obesity in comparison with control and obese reduction, and notably increased in obese reduction in comparison with control (all p<0.01).

### Protein and mRNA and expressions of apoptotic and anti-apoptotic biomarkers in LV myocardium at the End of study period (n=8 for each group)

**Figure 5:**
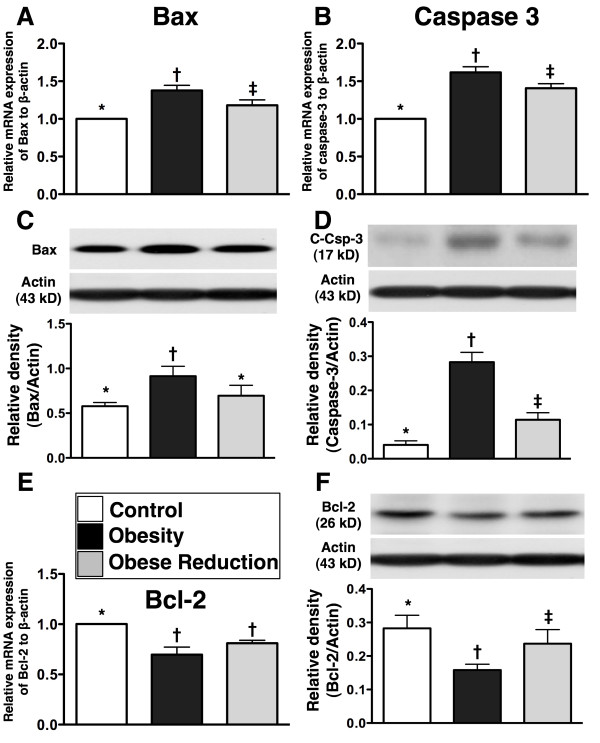
**Apoptic and anti-apoptotic biomarkers (n = 8 for each group). A) & B) **The mRNA expressions of Bax and caspase 3 were significantly higher in obesity group than in control and obese-reduction groups, and significantly higher in obese-reduction group than in control group. * vs. † vs. ‡, p<0.001. **C) **The Bax protein expression in mitochondria was notably higher in obesity group than in control and obese-reduction groups, but it displayed no difference between the later two groups. * vs. †, p<0.006. **D) **The cleaved caspase 3 protein expression was significantly higher in obesity group than in control and obese-reduction groups, and significantly higher in obese-reduction group than in control group. * vs. † vs. ‡, p<0.005. **E) & F) **showing the mRNA **E**) and protein (**F**) expressions of Bcl-2 were significantly lower in obesity group than in control and obese-reduction groups, and protein expression of Bcl-2 significantly lower in obese-reduction group than in control group. * vs. † vs. ‡, or * vs. †, p<0.008. All statistical analyses using one-way ANOVA, followed by Bonferroni multiple comparison post hoc test. Symbols (*, †, ‡) indicate significance (at 0.05 level) among **A) **to **F).**

To investigate the potential impact of obesity and obesity reduction on apoptosis, apoptotic and anti-apoptotic biomarkers were detected by RT-PCR and western blotting (Figure 5). Protein and mRNA expressions of Bax (mitochondrial) and caspase 3 (cleaved caspase 3 protein expression), two apoptotic biomarkers, were remarkably higher in obesity than in control and obese reduction, and the caspase 3 was notably higher in obese reduction than in control (all p<0.01), but Bax showed no difference between these two groups. Conversely, the mRNA and protein expressions of Bcl-2, an anti-apoptotic biomarker, were significantly lower in obesity than in control and obese reduction, and notably lower (only in protein level) in obese reduction than in control (p<0.008).

### Protein expressions of fibrotic and anti-fibrotic biomarkers in LV myocardium at the End of study period (n=8 for each group)

**Figure 6:**
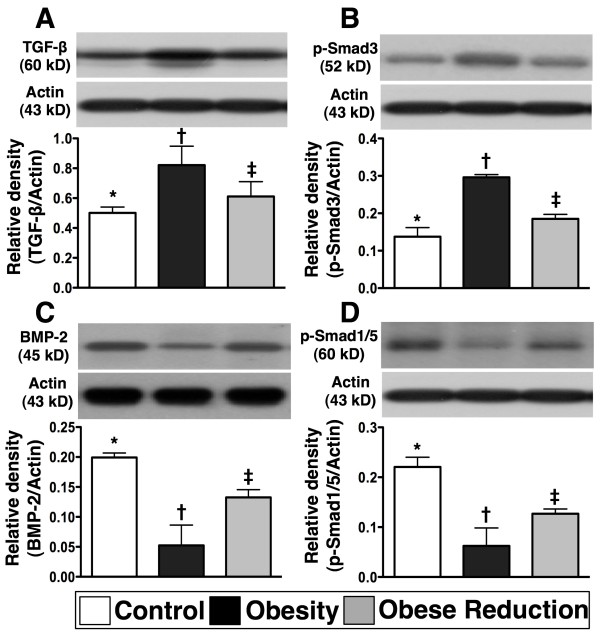
**The fibrotic and anti-fibrotic biomarkers (n = 8 for each group). A) & B) ** The protein expressions of transforming growth factor (TGF)-β and phosphorylated Smad3, two indicators of fibrosis, were significantly higher in obesity group than in control and obese-reduction groups, and notably higher in obese-reduction group than in control group. * vs. † vs. ‡, p<0.005. **C) & D) **The protein expressions of bone morphogenic protein (BMP)-2 and phosphorylated Smad1/5, two indices of anti-fibrosis, were remarkably lower in obesity than in control and obese-reduction groups, and significantly lower in obese-reduction group than in control group. * vs. † vs. ‡, p<0.001. All statistical analyses using one-way ANOVA, followed by Bonferroni multiple comparison post hoc test. Symbols (*, †, ‡) indicate significance (at 0.05 level) among **A) **to **D).**

To understand the influence of obesity and reduction in obesity on the protein expressions of fibrotic and anti-fibrotic markers in the LV myocardium, western blot analysis was performed (Figure 6). As expected, the protein expressions of TGF-β and phosphorylated Smad3, two indices of fibrosis, were significantly higher in obesity than in control and obese reduction, and significantly higher in obese reduction than in control (all p<0.005). In contrast to these findings, the protein expressions of BMP-2 and phosphorylated Smad1/5, two anti-fibrotic indicators, showed an opposite pattern to TGF-β and phosphorylated Smad3 in the three groups (p<0.001).

### Protein and mRNA expressions of anti-oxidant biomarkers in the LV myocardium at the End of study period (n=8 for each group)

**Figure 7:**
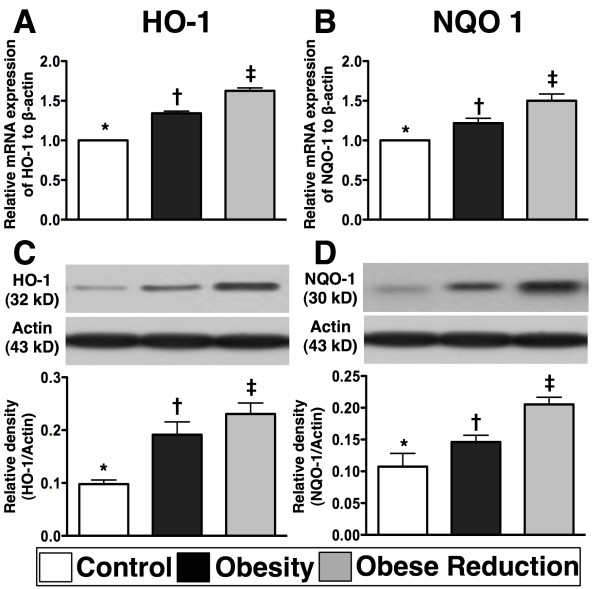
**The mRNA and protein expressions of anti-oxidant biomarkers (n = 8 for each group).** The mRNA (**A, B**) and protein (**C, D**) expressions of heme oxygenase (HO)-1 and NAD(P)H Quinone Oxidoreductase (NQO) 1 were significantly higher in obese-reduction group than in control and obesity groups, and significantly higher in obesity group than in control group. * vs. † vs. ‡, p<0.0001. All statistical analyses using one-way ANOVA, followed by Bonferroni multiple comparison post hoc test. Symbols (*, †, ‡) indicate significance (at 0.05 level) among **A) **to **D).**

To examine whether reduction in obesity could restore the anti-oxidant effect in the LV myocardium, anti-oxidant biomarkers were detected by RT-PCR and western blot (Figure 7). The results demonstrated that the mRNA and protein expressions of HO-1 and NQO-1, two indices of anti-oxidant cell response, were remarkably higher in obese reduction than in control and obesity, and significantly higher in obesity than in control (all p<0.0001).

## Discussion

This study investigated the role of obesity and subsequent reduction in obesity on heart function using a mouse model of obesity. Inflammation, oxidative stress, ROS, vasoconstriction, apoptosis and fibrosis in the LV myocardium were all attenuated by reduction in obesity. Further, obesity-impaired anti-oxidant expression and LV function were restored by reduction in obesity, and obesity-promoted LV remodeling was inhibited by reduction in obesity.

### Benefit of obesity reduction preserving heart function and inhibiting LV remodeling

The most important finding in this study was that compared with normal controls, LVEF and LV fractional shortening were significantly lower whereas LVEDD and LVESD, two indexes of LV remodeling, were notably higher in obese animals. Of particular importance, LV function was significantly preserved and LV remodeling was remarkably attenuated in animals following obesity/body-weight reduction.

It is well known that hyperglycemia and obesity are the two components of metabolic syndrome. Additionally, metabolic syndrome which is frequently found to be associated with the presence of dyslipidemia and insulin resistance that are the risk factors of atherosclerosis. The obese mice in the current study also exhibited the majority of these atherosclerotic risk factors. A previous study showed that poor DM control impaired LVEF [27]. However, the results of our study did not support the finding of poor DM control that ultimately impaired the LVEF function. Additionally, although echocardiography revealed that LV function was impaired in obese mice, the pathological findings (i.e., H.&.E stain) did not find any obstructive coronary artery disease. This finding suggests that other confounders rather than atherosclerotic obstructive coronary artery disease could play a crucial role in impairing heart function obese mice. Our suggestion may be supported by the fact that previous reports have stated that nearly 20% of DM patients are diagnosed with diabetic cardiomyopathy because of myocardial dysfunction and congestive heart failure (CHF) in the absence of coronary artery disease [28,29]. However, the mechanistic basis of myocardial dysfunction remains uncertain in setting of obesity.

### Impact of reduction in obesity in attenuating inflammation, oxidative stress and generation of vasoconstriction and ROS

Undoubtedly, increased oxidative stress, ROS and inflammatory processes which involve the endothelium and smooth muscle are essential in the development of all stages of atherosclerosis [14,18,19,30-33]. In addition, the association between obesity, inflammation and oxidative stress [13-15,34] and cardiovascular disease [9-13,15,16] is well documented. However, whether chronic inflammation and oxidative stress also take place in the LV myocardium is currently unclear, particularly, the occurrence is related to obesity-induced increases in oxidative stress and inflammation are unknown. One important finding of the present study was that as compared with normal controls, the ROS (gene and protein expressions of NOX-1 and NOX-2), inflammatory (protein expression of NF-κB and gene expressions of MMP-9, PAI-1 and leptin) and oxidative (oxidized protein) biomarkers in the LV myocardium were found to be substantially increased in obese mice. Additionally, vasoconstriction was increased and levels of ROS were higher in obese mice than in normal controls. In a previous study we found that inflammatory reactions enhanced the levels of ET-1 and generation of oxidative stress in the myocardium [35]. Our current findings, support the findings of this previous study [35], and further suggest interplay among inflammation, oxidative stress, generation of ROS and ET-1 in the obese myocardium. Importantly, these biomarkers were remarkably decreased following reduction in obesity. Enhanced inflammatory reaction and oxidative stress are associated with myocardial damage and deterioration of heart function [27,35]. Our findings could at least partially explain why LV function was impaired in obese mice and restored in obese mice after weight reduction.

### Role of reduction in obesity in ameliorating apoptosis and fibrosis and preserving anti-oxidant functions

As compared with normal controls, the apoptotic biomarkers (protein expressions of Bax and casapse 3) in LV myocardium were significantly enhanced in obese mice. On the other hand, the anti-apoptotic biomarker (protein expression of Bcl-2) in the LV myocardium was markedly suppressed in obese mice. Furthermore, an association between increased apoptotic and fibrotic biomarkers (protein expressions of TGF-β and p-Samd3) in the LV myocardium in obese mice was also found. Of importance, these parameters were notably decreased whereas the anti-fibrotic biomarkers (the protein expressions of BMP-2 and p-Smad1/5) were significantly increased in mice after reduction in obesity. In a rodent model of dilated cardiomyopathy, myocardial apoptosis and fibrosis were markedly enhanced in the myocardium in association with remarkably reduced LV function [20]. Our current findings compare with those findings [20], and may also partly explain why LVEF was significantly reduced and LV remodeling was markedly increased in obese animals.

HO-1 and NQO1 are markedly deceased in acute lung and kidney ischemia-reperfusion injury [36,37]. These two anti-oxidant biomarkers, whose expression was reversed after stem-cell therapy, are known to play an important role for against the renal parenchyma from ischemia-reperfusion injury [36,37]. A principal finding in the present study was that the gene and protein expressions of anti-oxidant biomarkers (i.e., HO-1 and NQO 1) in LV myocardium were significantly suppressed in obese mice and reversed in obese animals after body weight reduction. Hence, our findings, in addition to reinforcing the findings of those experimental studies [36,37], may also partially explain why markers of apoptosis and fibrosis and LV remodeling were significantly increased whereas LV function was notably reduced in obese mice, and these indicators were ameliorated after reduction in obesity.

### Study limitations

**Figure 8:**
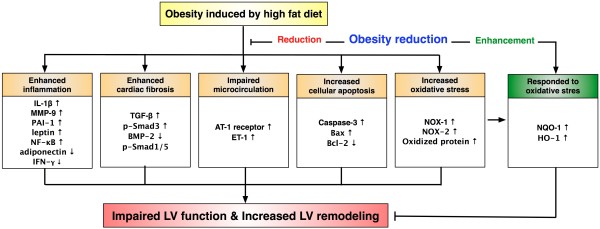
**Proposed molecular-cellular factors underlying the effects of obesity control (i.e., body weight reduction) on heart function in a mouse model based on the findings of the present study. **AT-1 = angiotensin II type 1; BMP-2 = bone morphogenic protein 2; ET = endothelin; HO = mheme oxygenase, IL-1 = interleukin 1, INF-γ = interferon-gamma; MMP-9 = matrix metalloproteinase; NF-κB=nuclear factor-κB; NOX = NADPH oxidase; NQO = NAD(P)H Quinone Oxidoreductase; PAI = plasminogen activator inhibitor; p-Smad1/5 = phosphorylated Smad1/5; p-Smad3 = phosphorylated Smad3; TGF-β = transforming growth factor beta.

This study has several limitations. First, this study did not address whether inflammation and oxidative stress was the cause of apoptosis and fibrosis in the LV myocardium of obese mice or vice versa. Second, another control group of weight-reduced with a high-fat diet did not performed in the current study. Thus, we did not completely exclude the possibility of confounding factor of diet composition that influenced the results of the present study. Third, we remain uncertain for why anti-oxidant markers were higher in obesity than control, and were highest in obese reduction. Perhaps, these findings suggest that inherent anti-oxidant system can be initiated by obesity in the LV myocardium in response to obesity-induced stress and that this effect can be further enhanced by reduction in obesity. The protein expressions of leptin and adiponectin receptors and the serum levels of these two biomarkers which would provide a more complete understanding on the mechanistic basis of obesity-related signaling pathway for LV dysfunction were not examined in the current study. Finally, although LV remodeling was significantly increased and LVEF was decreased in obese animals in comparison to animals in which obesity was reduced, we remain uncertain whether these ROS, inflammatory and oxidative-stress biomarkers directly participated in affecting the heart function. Overproduction of circulating aldosterone in setting of obesity has been reported by previous studies [38]. An increase in circulating level of this neuropeptide hormone, an indicator of renin-angiotensin-aldosterone system (RAAS), is undoubtedly harmful to the cardiovascular system. This perhaps could also patriotically explain the findings of our study. The molecular-cellular factors we propose to be involved in the observed changes are illustrated in Figure 8.

## Conclusion

This study demonstrated that in a mouse model, obesity enhanced inflammation, oxidative stress, apoptosis, fibrosis and LV remodeling, and impaired LV function; and all these traits could be reversed by a reduction in obesity. Our findings highlight the important role of obesity in damage to the myocardium, and show that such damage may be caused by factors other than those related to atherosclerotic-obstructive coronary artery disease.

## Competing interests

The authors declare that they have no competing interests.

## Authors’ contributions

All authors have read and approved the final manuscript. HTW, CFL, THT, and HWC designed the experiment, drafted and performed animal experiments. YLC, CYT , SL,YYZ, HTC, SYC, SC, and CHY were responsible for the laboratory assay and troubleshooting. HTW, CFL, and HKY participated in refinement of experiment protocol and coordination and helped in drafting the manuscript. All authors report no disclosures and have any commercial associations or interests, including consultancies, stock ownership or other competing equity interest.
